# Extraperitoneal vs. Transperitoneal Cesarean Section: A Systematic Literature Review of Safety and Outcomes

**DOI:** 10.3390/jcm15166153

**Published:** 2026-08-07

**Authors:** Daniel Wolder, Luka Velemir, Clémentin Castel, Anna Błażuk-Fortak, Agata Michalska, Katarzyna Kwas-Sarnacka, Grzegorz Świercz, Israel Hendler

**Affiliations:** 1 Department of Obstetrics and Gynecology, Jan Kochanowski University in Kielce, 25-369 Kielce, Poland; d.wolder@wp.pl (D.W.);; 2 Gynecology Institute of Nice—French Riviera, 06000 Nice, France; 3 Department of Obstetrics and Gynecology, Regional Hospital in Kielce, 25-736 Kielce, Poland; 4 Department of Surgical and Oncologic Gynecology, 1st Department of Gynecology and Obstetrics, Medical University of Lodz, 92-213 Lodz, Poland; 5 Department of Obstetrics and Gynecology, EMMS Nazareth Hospital, Nazareth 16100, Israel; 6 The Azrieli School of Medicine, Bar Ilan University, Safed 1311502, Israel

**Keywords:** cesarean section, extraperitoneal cesarean section, FAUCS, maternal outcomes, pain management, postoperative recovery, systematic review

## Abstract

**Background:** This systematic review aimed to evaluate the safety and outcomes of extraperitoneal cesarean section (EPCS), including the French Ambulatory Cesarean Section (FAUCS), by comparing them to those of transperitoneal cesarean section (TCS). **Data Sources**: The search was conducted using the following electronic bibliographic databases: PubMed, Web of Science, and Scopus. Studies were included if they were observational or interventional and investigated EPCS, including FAUCS, as the primary intervention. Exclusion criteria were animal studies, reviews, editorials, and conference abstracts. **Methods**: Two reviewers independently assessed the risk of bias using RoB 2 for RCTs and ROBINS-I for retrospective studies. Studies were grouped into comparable categories, and data were synthesized in summary tables and by comparative descriptive analysis. **Results**: In comparative studies, EPCS was associated with significantly lower rates of postoperative complications, such as fever (4% vs. 16% for TCS) and elevated C-reactive protein or leukocytosis (0% vs. 16–20%). EPCS reduced postoperative pain, with lower VAS scores at 24 h (e.g., 2.5 vs. 4.0 in TCS, *p* < 0.001) and shorter analgesic use. Time to return of bowel function was faster in EPCS and modified EPCS (6–8 h vs. 18–24 h in TCS, *p* < 0.01). Hospital stays were shorter after EPCS (4.15 ± 0.89 days vs. 4.82 ± 1.34 days, *p* = 0.027). FAUCS enabled early discharge within 24–48 h in 86–93% of cases and reduced morphine use (0.8% vs. 38% in TCS). Neonatal outcomes, including Apgar scores and cord pH, were similar across all groups. **Conclusions**: EPCS offers several potential advantages over TCS, including less postoperative pain, faster recovery, and shorter hospital stays. EPCS and its FAUCS variant are also associated with quicker return of bowel function and lower analgesic needs. TCS, while enabling faster fetal delivery, is linked to greater postoperative discomfort and slower recovery. However, current evidence is limited, and further high-quality trials are needed to confirm these benefits and assess long-term outcomes.

## 1. Introduction

Cesarean section is a critical intervention in obstetrics, providing a life-saving option for high-risk pregnancies where vaginal delivery would pose significant risks to the mother and/or baby [1]. The cesarean section is conventionally performed through low transverse abdominal wall opening followed by transperitoneal access to the lower uterine segment, which is then opened to deliver the baby. The rate of cesarean section is increasing worldwide and becoming a growing concern. Over 20 percent of all deliveries lead to cesarean section [2]. In the least developed countries, over 8% of women undergo cesarean section. In Europe, cesarean section rates exhibit considerable variation, ranging from as low as 17.4% in the Netherlands, Finland, and Norway to a high of 62.3% in Cyprus in 2023 [3,4] {https://in-cyprus.philenews.com/local/cyprus-tackles-soaring-caesarean-section-rates-with-financial-incentives, accessed on 17 July 2024}. The global cesarean section rate has increased from 7% in 1990 to 21% currently according to the WHO [5]. Projections suggest that by 2030, the prevalence of cesarean sections will reach 63% in Eastern Asia, 54% in the Caribbean, 50% in Western Asia, 47% in Southern Europe, 48% in Northern Africa, and 45% in New Zealand and Australia [5,6].

Compared with vaginal delivery, cesarean sections are associated with a higher incidence of morbidity for both mother and child, incurring substantial care and higher costs, as measured by the mean length of the hospital stay, use of analgesics, and potential complications, including adhesions, pelvic organ damage, placentation abnormalities, and uterine rupture [7,8]. By avoiding the opening of the abdominal cavity, extraperitoneal cesarean section (EPCS) offers several potential advantages over conventional transperitoneal cesarean section (TCS), primarily in postoperative recovery and pain management. Studies indicate that EPCS demonstrates an association with significantly lower postoperative pain scores and reduced analgesic requirements, with patients reporting less maximum surgical site pain compared with those undergoing TCS [9]. The potential benefits of EPCS are supported by several biological mechanisms. By avoiding entry into the peritoneal cavity, EPCS minimizes direct irritation of the peritoneum, which is richly innervated and highly sensitive to surgical trauma. Reduced manipulation of the bowel and peritoneal surfaces may decrease activation of the inflammatory cascade, resulting in lower postoperative cytokine release, earlier recovery of gastrointestinal motility, and less postoperative pain. Furthermore, limiting exposure of the peritoneal cavity may reduce bacterial contamination, decrease adhesion formation, and lower the risk of postoperative infectious complications. These mechanisms provide a plausible physiological explanation for the improved recovery reported in several clinical studies [10]. However, EPCS may result in a longer surgery as well as longer fetal extraction, which raises concerns in cases of acute fetal distress [11]. On the other hand, in women with a history of multiple cesarean sections, the potential for reduced intraperitoneal adhesions and lower infection rates makes EPCS an option worth considering [12].

The French Ambulatory Cesarean Section (FAUCS) is a specific method that employs an innovative extraperitoneal operative technique. It involves a paramedian vertical incision through the aponeurosis, a left paravesical extraperitoneal approach, a purse-string suture on the uterine wall, and skin closure using adhesive glue [13]. This technique was first described in the mid-1990s. Previous research has indicated that FACUS is associated with less postoperative pain; a shorter recovery time, with hospital discharge reported as occurring the day after surgery in 90% of cases; and a reduced requirement for pain relief medications. Additional advantages include decreased blood loss, lower post-surgical complication rates, and improved maternal outcomes [14].

Although there are indications of potential benefits associated with the use of EPCS, including the FAUCS method, high-level evidence supporting these findings is lacking. Current evidence remains limited because most available studies are single-center investigations with relatively small sample sizes, heterogeneous patient populations, different surgical techniques, inconsistent outcome measures, and varying perioperative protocols. Furthermore, only a limited number of randomized controlled trials have been published, making it difficult to draw firm conclusions regarding the comparative safety and effectiveness of EPCS. To date, no systematic reviews have been conducted to summarize the available evidence regarding this technique. Given the increasing number of comparative studies published in recent years and the absence of a comprehensive synthesis of the available evidence, a systematic review is needed to critically evaluate the current literature, identify consistent findings, assess study quality, and highlight remaining knowledge gaps.

We hypothesized that EPCS, including FAUCS, may improve postoperative maternal recovery and reduce postoperative morbidity compared with conventional transperitoneal cesarean section without adversely affecting maternal or neonatal safety. Therefore, the aim of this systematic review was to evaluate the available evidence regarding the safety and clinical outcomes of EPCS compared with conventional transperitoneal cesarean section.

## 2. Material and Methods

### 2.1. Study Design

This study is a systematic literature review conducted following the Preferred Reporting Items for Systematic Reviews and Meta-Analyses (PRISMA) guidelines, using predefined eligibility criteria, search strategy, and methods for study selection, data extraction, and risk-of-bias assessment [15]. The review protocol was prospectively registered in PROSPERO (registration number CRD420261363400).

The review was developed based on the PICO framework and aimed to answer the following research question: Among pregnant women undergoing cesarean section (Population), does extraperitoneal cesarean section, including FAUCS (Intervention), compared with conventional transperitoneal cesarean section or Misgav Ladach cesarean section (Comparator), improve maternal and neonatal outcomes (Outcomes)?

### 2.2. Eligibility Criteria

Studies were deemed eligible for inclusion based on predefined criteria. Inclusion criteria encompassed both observational and interventional studies investigating EPCS as the primary intervention or exposure, including the FAUCS as a specific method. Studies were excluded if they involved animal models, constituted review articles or editorials, included conference abstracts, or were published before the year 2000. Studies published before 2000 were excluded because contemporary cesarean section techniques, perioperative management, anesthesia protocols, antibiotic prophylaxis, and enhanced recovery pathways have substantially evolved over the last two decades. Earlier studies were considered less representative of current surgical practice and therefore of limited applicability to modern clinical decision-making. A literature search was conducted on 10 November 2024.

### 2.3. Information Sources

The search was conducted using the following electronic bibliographic databases: PubMed, Web of Science, and Scopus. Retrieved studies were subsequently screened and analyzed based on the predefined eligibility criteria.

### 2.4. Search Strategy

The search strategy for this review was developed based on the PICO framework to ensure a systematic and comprehensive approach [16]. Terms related to the study population included “pregnancy” and “pregnant woman.” Intervention-related terms comprised “extraperitoneal cesarean section,” “French Ambulatory Cesarean Section,” “French Ambulatory Cesarean,” and “FAUCS.” Outcome-related terms focused on “safety,” “complications,” “mortality,” as well as “maternal outcomes” and “neonatal outcomes”. Search strings are shown in Table 1.

### 2.5. Selection Process

**Figure 1:**
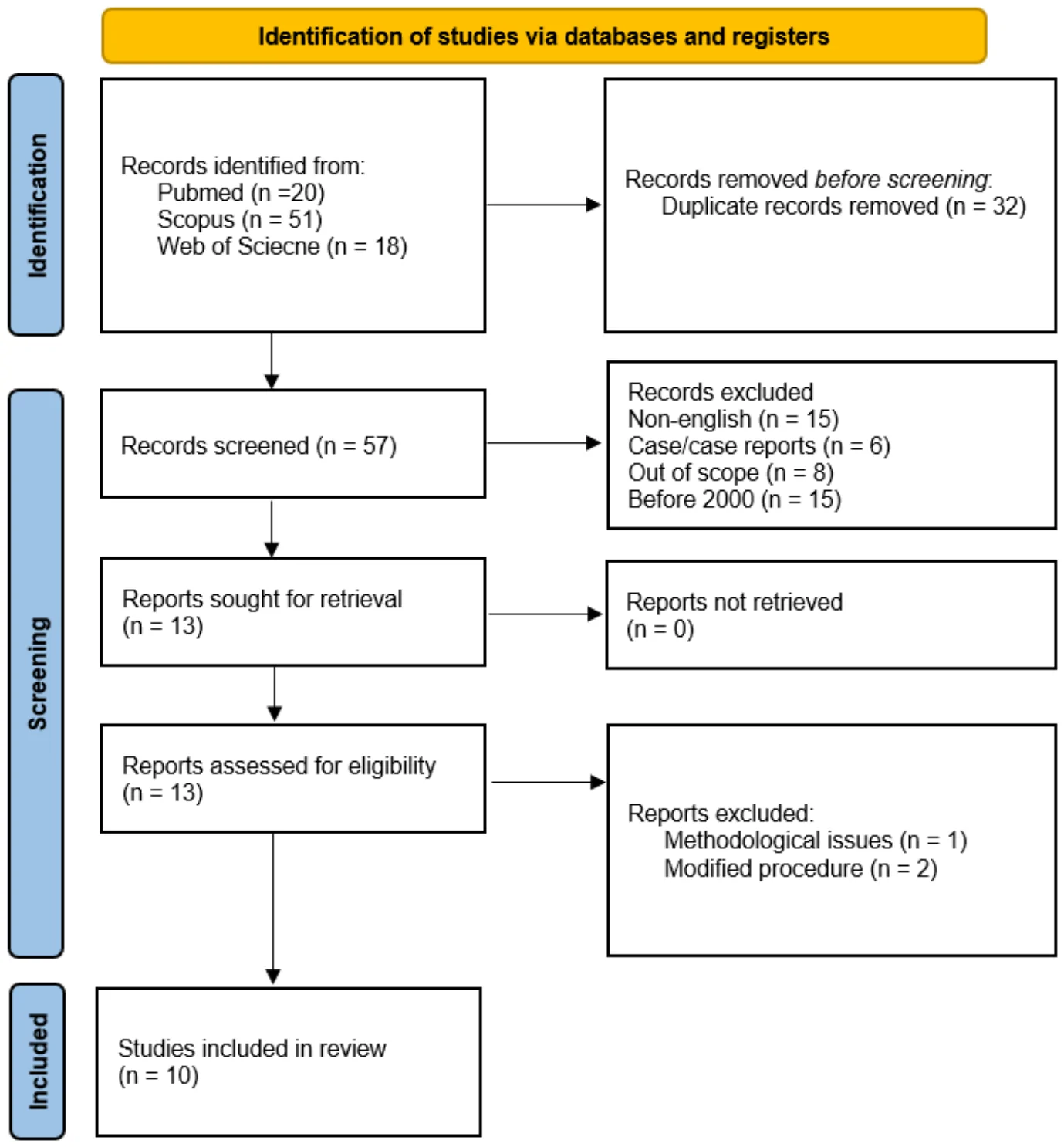
PRISMA Flowchart.

After database searching, all retrieved records were exported into reference management software (Mendeley Reference Manager v2.148.0), and duplicate records were removed before screening. Titles and abstracts were independently assessed by two reviewers according to the predefined eligibility criteria. Studies considered potentially eligible underwent full-text evaluation by the same reviewers. Any disagreement regarding study eligibility was resolved through discussion with a third reviewer. The study selection process is summarized in the PRISMA flow diagram in Figure 1.

A total of 89 records were identified from PubMed (20), Scopus (51), and Web of Science (18). After removing 32 duplicates, 57 unique records were screened. Following the initial screening, 44 records were excluded due to various reasons: 15 were non-English, six were case reports, eight were out of scope, and 15 were published before the year 2000.

Thirteen full-text articles were assessed for eligibility. One study was excluded for methodological reasons (retrospective design with ‘random division’ of participants) [17]. Two further records were excluded because they examined modified procedures (Modified FAUCS vs. TCS; Modified Extraperitoneal Cesarean Section vs. TCS) that did not align with our predefined comparators [10,14]. The final synthesis included nine studies: EPCS vs. TCS (*n* = 4), FAUCS vs. TCS (*n* = 4), and FAUCS vs. Misgav Ladach Cesarean Section (MLCS) (*n* = 2). PRISMA checklist available in the Supplementary Materials.

### 2.6. Data Extraction

A standardized data extraction sheet was used to collect relevant information from the included studies. Extracted data included authors’ names, year of publication, study design, sample size, study groups, and reported clinical outcomes. For studies comparing specific outcomes, data were extracted in greater detail, focusing on comparative measures such as time to fetal delivery, operative time, blood loss, recovery metrics, and neonatal outcomes. This detailed extraction allowed for the organization of outcome-specific data across studies. One reviewer conducted the extraction to ensure reliability, while another verified the data for accuracy. The extracted data were organized into structured tables to facilitate comparative analysis and synthesis.

### 2.7. Study Risk-of-Bias Assessment

The risk of bias was assessed separately for randomized controlled trials (RCTs) and retrospective studies using appropriate tools for each study design. For RCTs, the Cochrane Risk of Bias 2.0 (RoB 2) tool was employed. This tool evaluates bias across five domains: randomization process, deviations from intended interventions, missing outcome data, measurement of outcomes, and selection of reported results [18]. Each domain was rated as low risk, with some concerns, or high risk of bias, with an overall judgment derived from the individual domain ratings [19]. The Risk of Bias In Non-randomized Studies of Interventions (ROBINS-I v2) tool was used for retrospective studies. This tool evaluates bias across seven domains: confounding, participant selection, classification of interventions, deviations from intended interventions, missing data, measurement of outcomes, and selection of reported results [20]. Each domain was assessed as having a low, moderate, serious, or critical risk of bias, with the overall risk determined by the highest risk category across domains. Two reviewers independently conducted the risk-of-bias assessment, with disagreements resolved through discussion or consultation with a third reviewer. The robvis app was used for the visualization of results [21].

### 2.8. Assessment of Publication Bias

Because of the limited number of included studies, substantial clinical and methodological heterogeneity, and the absence of quantitative synthesis, a formal assessment of publication bias (e.g., funnel plot analysis or tests for small-study effects) was not performed. To minimize the risk of publication bias, a comprehensive search was conducted in three electronic databases, and the reference lists of eligible studies were screened for additional relevant publications. Gray literature and unpublished studies were not systematically searched, which may have increased the risk of publication bias and should be considered when interpreting the findings.

## 3. Results

### 3.1. Study Characteristics

The included studies comprised five randomized controlled trials (RCTs), two retrospective studies (one cohort and one case–control), three prospective cohort studies, and two retrospective descriptive studies. The research was conducted across various countries, including Austria, Turkey, Croatia, Israel, France, Poland and Tunisia, reflecting a wide geographic distribution. The studies’ publication years range from 2013 to 2025.

The studies investigated a variety of interventions, including comparisons of extraperitoneal cesarean section (EPCS) with transperitoneal cesarean section (TCS) and the French Ambulatory Cesarean Section (FAUCS) with TCS or Misgav Ladach Cesarean Section (MLCS). The designs ranged from blinded and open-label randomized trials to retrospective descriptive analyses, highlighting differences in study rigor and methodology. This diversity of study types and settings provides a broad basis for analyzing the outcomes of these surgical techniques.

### 3.2. Risk of Bias in Studies

**Figure 2:**
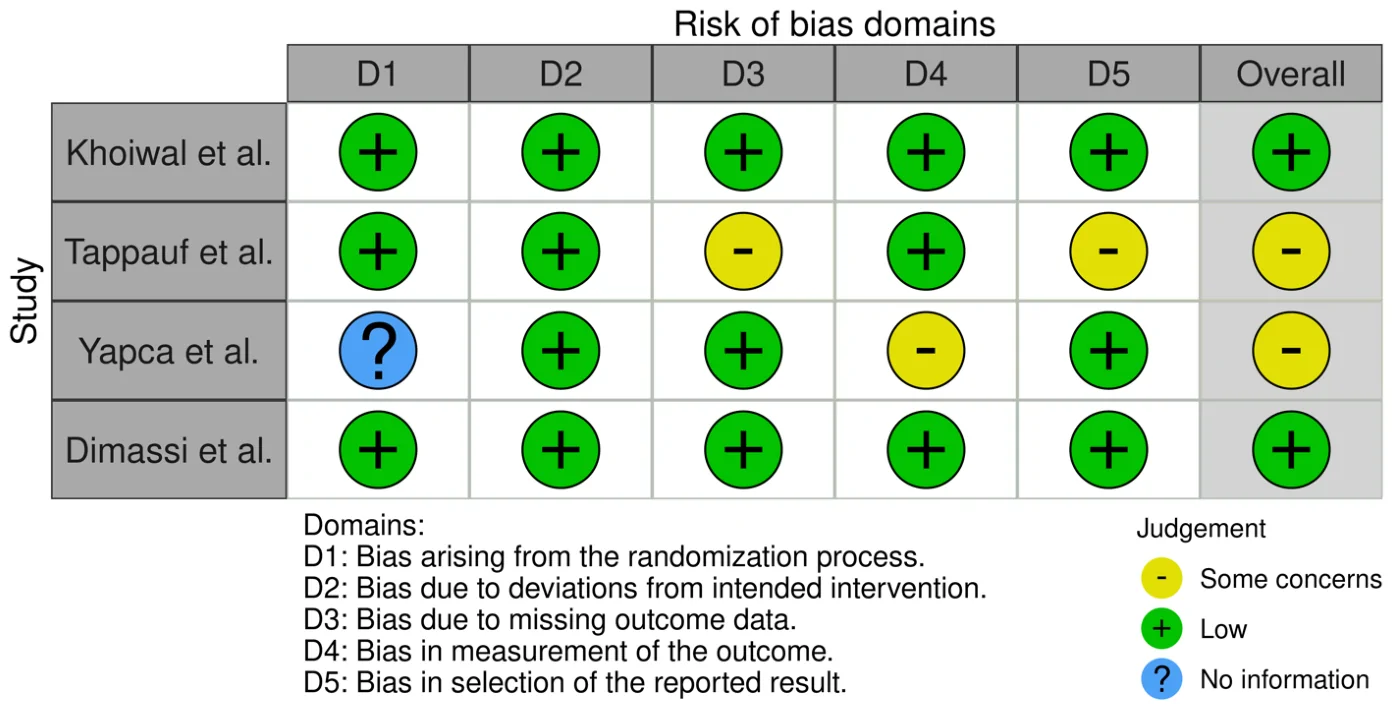
Risk-of-bias assessment using RoB 2 Tool for included RCT studies [9,11,22,23].

The risk-of-bias assessment was conducted separately for randomized controlled trials (RCTs) and observational studies due to differences in assessment methodologies. Khoiwal et al. and Dimassi et al. demonstrated a low risk of bias across all assessed domains, indicating strong methodological rigor [11,22]. In contrast, Tappauf et al. and Yapca et al. were judged to have some concerns in specific domains [9,23]. Tappauf et al. exhibited potential bias due to missing outcome data (D3) and selection of the reported result (D5), while Yapca et al. raised concerns about outcome measurement (D4) and provided insufficient information on the randomization process (D1) [9,23]. This uncertainty in Yapca et al. may affect the reliability of their findings [23]. Overall, while two studies had a low risk of bias, the remaining studies had methodological limitations, particularly in data completeness, outcome measurement, and reporting practices, which should be considered when interpreting the results (Figure 2).

Findings from randomized controlled trials with a low risk of bias were considered to provide stronger evidence than retrospective observational studies, particularly regarding postoperative pain, recovery time, and neonatal outcomes. Conversely, findings from retrospective descriptive studies should be interpreted with greater caution because of their greater susceptibility to confounding and selection bias.

**Figure 3:**
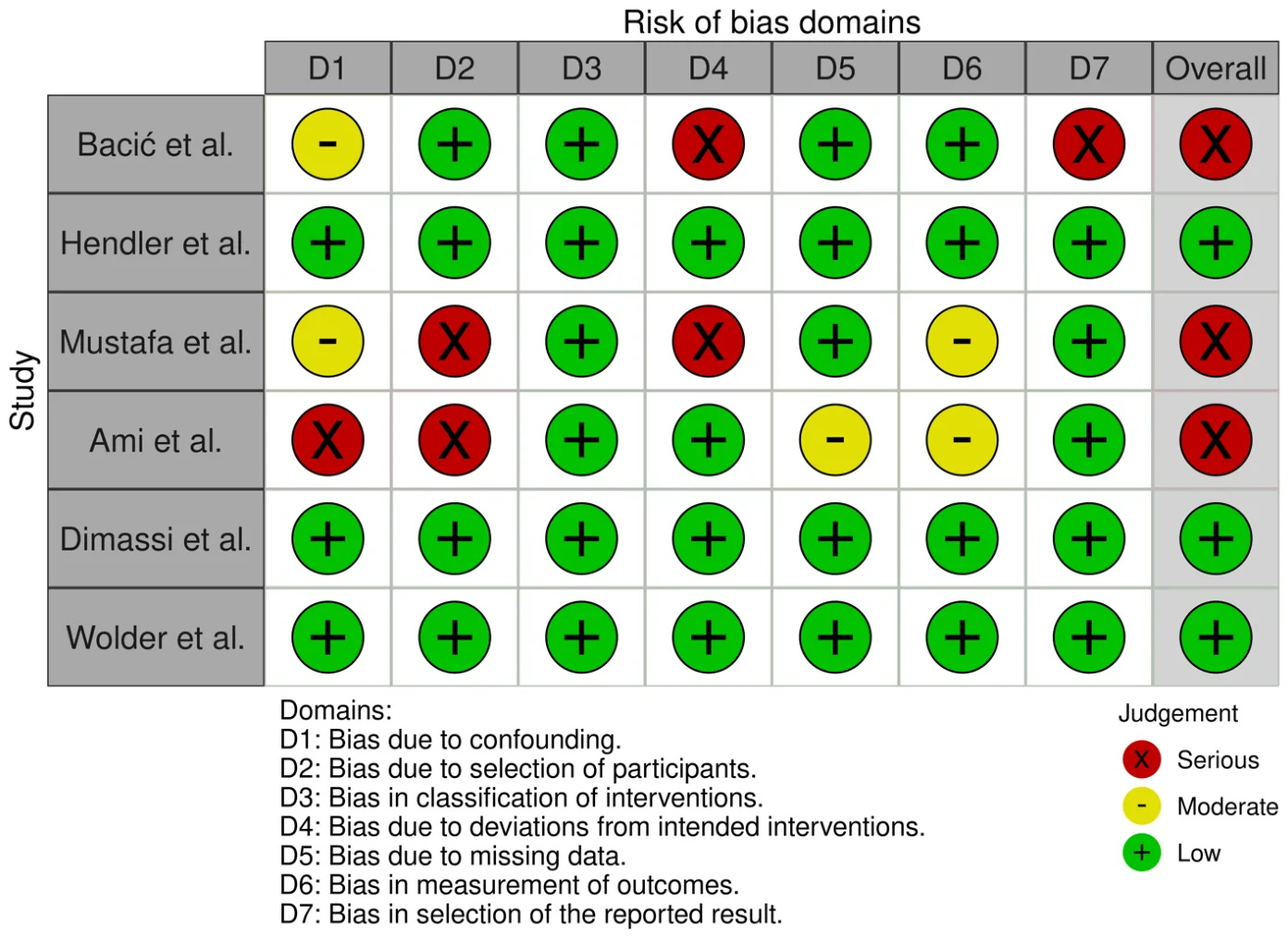
Risk-of-bias assessment using ROBINS-I v2 Tool for included observational studies [12,13,22,25,26,27].

Studies by Dimassi et al. and Wolder et al. demonstrated a low risk of bias across all domains, indicating strong methodological rigor [24,25]. Hendler et al. also exhibited a generally low risk of bias, with only moderate concerns in missing data (D5) and outcome measurement (D6) [26]. In contrast, Bacić et al. and Mustafa et al. showed a moderate risk of bias due to confounding (D1), while Mustafa et al. additionally had serious concerns regarding participant selection (D2) and deviations from intended interventions (D4), leading to an overall serious risk of bias [12,27]. Ami et al. exhibited the highest risk, with serious bias in confounding (D1), participant selection (D2), and outcome reporting (D7), resulting in an overall judgment of serious risk of bias [13]. These findings suggest that while some studies maintain methodological robustness, others exhibit substantial limitations, particularly in confounding, participant selection, and adherence to intended interventions, which must be considered when interpreting their results (Figure 3).

### 3.3. Results of Studies

#### 3.3.1. EPCS vs. TCS—Retrospective and RCT Studies

This section summarizes the findings of four studies comparing EPCS with TCS, including one retrospective study and three randomized controlled trials (RCTs). Bacić et al. performed a retrospective case–control study involving 178 patients, 88 of whom underwent EPCS and 90 underwent TCS [12]. The groups were matched for age and pre-pregnancy body mass index to ensure comparable baseline characteristics. Khoiwal et al. conducted an open-label randomized controlled trial involving 68 women, 34 in the EPCS group and 34 in the TCS group [11]. The study aimed to determine whether the extraperitoneal technique was superior to the transperitoneal method in terms of time to delivery, intraoperative outcomes, and postoperative recovery, including restoration of bowel function and pain levels. Tappauf et al. performed a prospective, patient-blinded randomized trial with 54 participants, equally divided between the EPCS and TCS groups, to test the hypothesis that the EPCS technique reduces postoperative pain without increasing intraoperative or postoperative complications [9]. Yapca et al. conducted the largest randomized trial, involving 210 pregnant women, with 105 undergoing EPCS and 105 undergoing TCS [23]. This study specifically compared the two techniques regarding the time to fetal delivery. Detailed characteristics of studies are shown in Table 2.

The study by Bačić et al. found no statistically significant differences between the EPCS and TCS groups in Apgar scores, erythrocyte counts, hemoglobin levels, or hematocrit values [12].

However, the study demonstrated that EPCS was associated with a lower incidence of postoperative complications. On the third postoperative day, none of the patients in the EPCS group had elevated C-reactive protein levels or leukocytosis, whereas in the TCS group, these were observed in 16% and 20% of patients, respectively. Similarly, only 4% of patients in the EPCS group had fever exceeding 38 °C on the third postoperative day, compared with 16% in the TCS group. Additionally, patients who underwent EPCS reported less postoperative pain and required fewer analgesics than those in the TCS group.

The comparison of outcomes between EPCS and TCS across randomized controlled trials is summarized in Table 3. Across the studies, TCS was generally associated with faster time to fetal delivery. For example, Khoiwal et al. reported a significantly shorter delivery time (from skin incision) in the TCS group (9.38 ± 1.83 min) compared with the EPCS group (14.26 ± 1.26 min; *p* < 0.001), whereas Yapca et al. and Tappauf et al. did not observe statistically significant differences [9,11,23]. Conversely, EPCS demonstrated shorter or comparable total operation times, with Tappauf et al. reporting significantly shorter EPCS times (28.9 ± 3.9 min) than TCS (34.7 ± 10.5 min; *p* = 0.012) [9].

Blood loss did not significantly differ between EPCS and TCS in Khoiwal et al.’s study, and other trials did not report this outcome [11]. EPCS was consistently associated with faster return of bowel function, as noted by Khoiwal et al. and Yapca et al. [11,23]. Neonatal outcomes, including Apgar scores at 1 and 5 min and umbilical cord pH, showed no significant differences between the techniques, with similar findings reported by Khoiwal et al., Tappauf et al., and Yapca et al. [9,11,23].

Regarding hemoglobin change, Khoiwal et al. observed a significantly smaller reduction in the EPCS group (1.04 ± 0.51 g/dL) compared with the TCS group (1.40 ± 0.40 g/dL; *p* = 0.001), whereas other studies, including Yapca et al., did not find significant differences. [11,23] Finally, hospital stay tended to be shorter or comparable for EPCS, with Khoiwal et al. reporting a significantly shorter duration for EPCS (4.15 ± 0.89 days) than for TCS (4.82 ± 1.34 days; *p* = 0.027) [11]. These findings suggest that EPCS may provide advantages in recovery time and postoperative outcomes while maintaining comparable safety and neonatal outcomes, as highlighted by multiple authors.

All three RCTs—Khoiwal et al., Tappauf et al., and Yapca et al.—compared postoperative pain and analgesic use between EPCS and TCS. However, pain measurement and reporting methods varied [9,11,23]. Khoiwal et al. used the visual analog scale (VAS) at 6, 12, and 18 h postoperatively; Tappauf et al. employed the numerical rating scale to assess peak pain on the first postoperative day; and Yapca et al. used VAS at 24 and 48 h. Despite these methodological differences, all studies reported lower postoperative pain in the EPCS group [9,11,23]. Khoiwal et al. and Tappauf et al. found statistically significant reductions in pain scores, and Yapca et al. observed lower VAS scores in the EPCS group at 24 and 48 h. Regarding analgesic use, EPCS was associated with significantly shorter analgesic duration in Khoiwal et al.’s study, reduced NSAID and opioid consumption in Tappauf et al., and lower oral and intravenous analgesic requirements in Yapca et al. [9,11,23]. Although differences in pain measurement complicate direct comparisons, the consistent trend of reduced pain and analgesic use in EPCS groups suggests that EPCS may offer advantages in postoperative pain management. Standardized methodologies in future studies could provide stronger evidence to support these findings.

Additionally, all three RCTs examined the incidence of nausea and vomiting, with EPCS generally showing a lower incidence of these symptoms than TCS. Khoiwal et al. reported no postoperative nausea or vomiting in the EPCS group, whereas 11.8% of TCS patients experienced nausea; however, the data collection period was not specified [11]. Tappauf et al. focused on intraoperative nausea and vomiting, observing these symptoms in three TCS patients but none in the EPCS group, a statistically significant difference (*p* < 0.001) [9]. Yapca et al. collected both intraoperative and postoperative data and found no significant differences in intraoperative nausea (four EPCS vs. five TCS patients) or vomiting (two TCS patients only) [23]. Postoperatively, nausea was slightly less common in the EPCS group at both 24 and 48 h, though the differences were not statistically significant. Vomiting after surgery was observed only in the TCS group at 24 h. These findings suggest that TCS may be associated with a higher risk of nausea and vomiting compared with EPCS. However, methodological differences in how these symptoms were measured and reported limit direct comparability and the strength of conclusions.

The above analysis highlights that the results of the three RCTs are not entirely consistent. For instance, while Khoiwal et al. and Yapca et al. found that EPCS was associated with a longer time to fetal delivery, Tappauf et al. observed the opposite trend, though the difference was not statistically significant [9,11,23]. Similarly, findings on total operation time and hemoglobin change varied across studies. It is also important to note the limitations of each trial. For example, Khoiwal et al.’s study had a small sample size, whereas Yapca et al. did not report blood loss [11,23]. These discrepancies and limitations underscore the need for further standardized, large-scale research to clarify the comparative advantages of EPCS and TCS.

#### 3.3.2. FAUCS Studies—Retrospective, Prospective and RCTs

The studies on FAUCS, as outlined in Table 4, include one comparative study and two descriptive studies without a comparison group. Hendler et al. [26] conducted a retrospective cohort study comparing 810 FAUCS cases with 217 TCS cases, focusing on surgical and postoperative outcomes, including total surgery time, maternal complications, and neonatal outcomes. In contrast, Mustafa et al. [27] performed a retrospective descriptive study of 50 FAUCS cases without a comparator, primarily evaluating surgical efficiency, maternal complications, and patient satisfaction. Similarly, Ami et al. [13] conducted a large retrospective descriptive study of 3441 FAUCS cases, assessing parameters such as surgical outcomes, hospital stay duration, and postoperative pain management. While Hendler et al. [26] provided comparative insights, the other two studies offered valuable descriptive data on FAUCS outcomes, emphasizing the need for more comparative research (Table 4). Due to methodological differences, including the lack of a comparator in two studies, the results cannot be directly compared and will be analyzed separately.

In addition, Wolder et al. conducted a prospective comparative cohort study that included 66 women (30 FAUCS vs. 36 conventional cesarean sections) and directly compared maternal and neonatal outcomes [25]. FAUCS was associated with significantly faster postoperative recovery, including earlier mobilization (6.0 ± 1.5 vs. 16.9 ± 6.7 h), earlier oral food intake (7.2 ± 1.6 vs. 17.0 ± 6.8 h), and earlier initiation of breastfeeding (3.3 ± 1.4 vs. 7.0 ± 5.5 h), all with *p* < 0.001. The use of weak opioids was significantly lower in the FAUCS group (56.7% vs. 80.6%, *p* = 0.036). Operative time was slightly longer in FAUCS (30.7 ± 4.1 vs. 26.4 ± 10.3 min, *p* = 0.006), while blood loss, rates of bladder injury, postpartum hemorrhage, and transfusion were comparable between groups. Neonatal outcomes, including Apgar scores, NICU admission, and birthweight, did not differ significantly, although umbilical cord arterial pH was slightly lower in the FAUCS group (7.33 ± 0.07 vs. 7.36 ± 0.06, *p* = 0.009), remaining within the physiological range. The authors concluded that FAUCS improves early maternal recovery without compromising maternal or neonatal safety [25].

Hendler et al. reported comparable overall surgical complications between the FAUCS (1.97%) and TCS (1.85%) groups, and specific complications, including bladder injury (0.1%), bowel injury (0.1%), blood transfusion (1.35%), and postpartum hemorrhage (1%), aligned with rates reported in the literature. Neonatal outcomes, including neonatal acidemia and NICU admissions, were similar across groups and consistent with favorable benchmarks in prior studies. Notably, analgesia use differed significantly, with only 0.8% of women in the FAUCS group requiring morphine, compared with 38% in the TCS group [26]. Additionally, recovery was significantly faster in the FAUCS group, with 86% achieving autonomy and opting for early discharge within 48 h, compared with 17% in the TCS group.

The mean total operation time reported by Ami et al. was 23 min, while Mustafa et al. reported a longer mean duration of 53.26 ± 11.62 min [13,27]. In Mustafa et al.’s study, surgical time decreased with experience, from 58.26 ± 12.25 min for the first 15 cases to 51.17 ± 9.73 min for subsequent procedures, underscoring the learning curve for different C-section methods. Postoperative pain was minimal in both studies. Mustafa et al. reported a mean 24 h VAS score of 1.08 ± 0.84 (on a 10-point scale), and Ami et al. noted that 2.9% of patients required morphine, while 20.9% required no painkillers. Regarding recovery, Ami et al. found that 92.94% of patients were eligible for hospital discharge within 24 h, with an average discharge time of 1.3 days. Similarly, Mustafa et al. observed that 44% of women were able to mobilize and urinate spontaneously within 4–6 h postoperatively. Both studies reported low rates of complications. Ami et al. observed 0.3% bladder injuries, 0.2% retropubic hematomas, and 0.1% parietal abscesses, with complications primarily linked to the training phase of surgeons or severe adhesions. Mustafa et al. reported one case each of bladder injury, endometritis, and incisional hematoma. Neonatal outcomes were favorable in both studies. Mustafa et al. reported a 6% rate of cord pH < 7.2 and 11.3% cases of vacuum-assisted fetal extraction. Ami et al. reported comparable rates of neonatal complications to other cesarean techniques but did not provide detailed neonatal metrics. In Mustafa et al.’s study, maternal satisfaction was high, and 94% of the women would recommend FAUCS to others [13,27].

#### 3.3.3. FAUCS vs. The Misgav Ladach Cesarean Section

Two studies comparing the FAUCS to the Misgav Ladach Cesarean Section (MLCS), both conducted by the research team led by Dimassi, reflect a progressive approach to evaluating this technique [22,24]. The first study, a prospective cohort study, focused on a preliminary assessment of FAUCS’s safety and effectiveness compared with the traditional MLCS method. The second study, conducted later, was a randomized controlled trial, enabling a more precise comparison between the two methods, particularly regarding postoperative pain and maternal autonomy. The characteristics of these studies are shown in Table 4.

Both studies by Dimassi et al. (2020 and 2021) showed that women after FAUCS reported significantly lower postoperative pain scores than after MLCS. Pain was measured every 6 h during the first 24 h after surgery [22,24]. In the 2020 study, the median VAS pain score on day 1 was 3 (IQR 2–5) for FAUCS vs. 4 (IQR 3.7–5) for MLCS (*p* < 0.001). In the 2021 trial, the mean pain score (PMPS) over 24 h was 1.87 for FAUCS vs. 2.93 for MLCS (*p* < 0.001), with the biggest difference at 6 h post-op (2.28 vs. 7.00). Recovery of functional autonomy was faster in the FAUCS group, with the 2021 study introducing “autonomy” as a composite variable that included standing, eating, and urinating. This study found that 94% of FAUCS patients achieved autonomy within 12 h, compared with only 4% in the MLCS group (*p* < 0.001). Additionally, both studies reported shorter hospital stays among FAUCS patients, with this difference statistically significant in both analyses (*p* < 0.001). In the 2020 study, the median hospital stay was 1 day (IQR 1–2) for FAUCS versus 2 days (IQR 2–3) for MLCS. In the 2021 trial, the mean length of stay was 1.18 ± 0.07 days for FAUCS versus 1.88 ± 0.05 days for MLCS. Importantly, no adverse effects on neonatal outcomes were observed in either study, as Apgar scores, infection rates, and readmission rates were comparable between groups. The 2021 study further evaluated umbilical cord pH and neonatal hospitalization rates, finding no significant differences between FAUCS and MLCS.

Despite these consistent findings, the studies differed in certain outcomes. The 2020 study associated FAUCS with longer operation times (*p* < 0.001), reporting a median duration of 50 min (IQR 40–60) for FAUCS versus 35 min (IQR 30–40) for MLCS. In contrast, the 2021 study reported no significant difference, with mean operation times of 43.3 ± 7.3 min for FAUCS and 38.4 ± 2.2 min for MLCS (*p* = 0.414), attributing this to the learning curve during the early phase of FAUCS implementation. Similarly, the 2020 study reported a higher frequency of instrument use for fetal extraction in the FAUCS group (*p* < 0.001), whereas the 2021 study acknowledged instrument use in 84% of FAUCS cases but provided no detailed comparison. Methodological differences between the studies also warrant attention. In the 2020 study, morphine was used in spinal anesthesia for both FAUCS and MLCS groups, whereas in 2021, morphine was used only in the MLCS group. The authors noted that this discrepancy may have influenced outcomes related to postoperative pain and recovery times.

## 4. Discussion

The review demonstrated that EPCS, particularly FAUCS, is associated with reduced postoperative pain, faster recovery, lower analgesic requirements, and a quicker return of bowel function. Compared with TCS, EPCS may also be linked to a shorter hospital stay. On the other hand, TCS is often associated with a shorter fetal delivery time. Studies on FAUCS indicate similar complication rates to TCS but with lower analgesic consumption and faster recovery.

Interpretation of the present findings should consider the substantial heterogeneity among the included studies. Considerable differences existed in study design, sample size, inclusion criteria, indications for cesarean section, surgeon experience, anesthesia protocols, perioperative management, and outcome assessment methods. These factors likely contributed to the variability observed across studies and limited the direct comparability of the reported outcomes. Formal statistical assessment of heterogeneity was not performed because the substantial clinical and methodological heterogeneity of the included studies precluded quantitative synthesis. Consequently, the findings should be interpreted as a qualitative synthesis of the available evidence rather than as pooled estimates of treatment effect.

Several factors may explain the discrepancies observed across studies. First, surgeon experience varied substantially, particularly for FAUCS, for which a pronounced learning curve has been described. Second, studies included both elective and emergency cesarean sections, which may have influenced operative time and neonatal outcomes. Third, perioperative analgesic protocols and anesthesia techniques differed considerably between centers. Finally, outcome definitions and measurement methods, particularly for postoperative pain and bowel recovery, were not standardized.

Among randomized controlled trials, postoperative pain represents the most consistent finding favoring EPCS. Despite differences in pain assessment methods and measurement timing, all randomized studies demonstrated lower postoperative pain scores and reduced analgesic requirements following EPCS compared with TCS. Earlier recovery of bowel function was also consistently reported across comparative studies. These findings are biologically plausible because avoidance of peritoneal entry reduces peritoneal irritation and bowel manipulation, thereby limiting postoperative inflammatory response and facilitating gastrointestinal recovery.

Conversely, evidence regarding operative characteristics remains less consistent. Although TCS generally resulted in shorter fetal extraction time, differences were not statistically significant in all studies. Similarly, total operative time varied considerably across studies, with some reporting shorter EPCS procedures and others reporting comparable or longer operative durations. These inconsistencies are likely explained by differences in surgeon experience, learning curves, patient selection, and surgical technique rather than by the surgical approach itself. Importantly, neonatal outcomes, including Apgar scores, umbilical cord pH, and neonatal intensive care unit admission, remained comparable across studies, suggesting that the potential maternal benefits of EPCS are not accompanied by clinically relevant neonatal disadvantages.

The available evidence regarding FAUCS further supports the potential benefits of extraperitoneal techniques. Comparative studies consistently demonstrated reduced postoperative analgesic requirements, earlier mobilization, faster return to oral feeding, shorter hospitalization, and more rapid recovery of maternal autonomy. Prospective comparative data have strengthened these observations by confirming faster postoperative recovery without an increase in maternal or neonatal complications. Nevertheless, operative time appears to depend strongly on surgical experience, emphasizing the importance of adequate training before widespread implementation of this technique.

The present review also highlights several important methodological limitations within the available literature. Although five randomized controlled trials were identified, most studies were single-center investigations with relatively small sample sizes. Observational studies varied considerably in patient selection and perioperative management, increasing the risk of confounding. Furthermore, differences in comparator groups, outcome definitions, and follow-up periods limited direct comparison between studies. Although a quantitative synthesis was initially considered, a meta-analysis was deemed inappropriate due to the limited number of studies, heterogeneous study designs, different comparator groups, variable outcome definitions, inconsistent pain assessment methods, and differences in perioperative protocols. Therefore, the conclusions of the present review should be interpreted with caution and primarily as hypothesis-generating rather than as definitive estimates of treatment effect.

### 4.1. Clinical Implications

The present findings suggest that EPCS and particularly FAUCS may be considered in centers with adequate surgical expertise, especially for elective cesarean deliveries where faster postoperative recovery, reduced pain, and earlier mobilization are desirable. However, due to the learning curve and limited high-quality evidence, widespread implementation should be accompanied by appropriate surgeon training and further prospective evaluation.

### 4.2. Future Research

Future multicenter randomized controlled trials with standardized surgical protocols are needed to compare EPCS, FAUCS, and conventional transperitoneal cesarean section. Future studies should include homogeneous patient populations, clearly defined indications for cesarean delivery, standardized pain assessment methods, long-term maternal outcomes, neonatal outcomes, cost-effectiveness analyses, and evaluation of the learning curve associated with these techniques.

## 5. Strengths and Limitations

This systematic review provides the first comprehensive overview of the available evidence regarding EPCS, including FAUCS, by synthesizing data from comparative and observational studies. The review included randomized controlled trials, prospective cohort studies, and retrospective studies, enabling a broad evaluation of maternal and neonatal outcomes across diverse clinical settings. More recently, prospective comparative studies have strengthened the available evidence supporting FAUCS.

However, several limitations should be acknowledged. These limitations should be considered when interpreting the clinical applicability of the present findings. The overall certainty of evidence was not formally assessed using the GRADE approach. Because quantitative synthesis was deemed inappropriate, pooled effect estimates such as relative risks, odds ratios, mean differences, and confidence intervals could not be calculated. Consequently, the conclusions are based on qualitative narrative synthesis rather than quantitative evidence. In addition, the available evidence is limited by the relatively small number of studies, considerable clinical and methodological heterogeneity, predominance of single-center investigations, variability in outcome definitions and perioperative protocols, and the inability to formally evaluate publication bias. Therefore, despite the encouraging findings, the current body of evidence remains limited, and large, well-designed multicenter randomized controlled trials are needed to confirm the observed benefits of EPCS and FAUCS.

## 6. Conclusions

The available evidence suggests that extraperitoneal cesarean section (EPCS), including French Ambulatory Cesarean Section (FAUCS), may offer several maternal benefits compared with conventional transperitoneal cesarean section. Across the included studies, EPCS was consistently associated with reduced postoperative pain, lower analgesic requirements, earlier recovery of bowel function, and shorter hospital stay, while maternal and neonatal safety outcomes appeared comparable to those of conventional techniques.

However, the available evidence is limited by substantial clinical and methodological heterogeneity, relatively small sample sizes, and the predominance of single-center studies. Consequently, the observed benefits should be interpreted cautiously, and definitive conclusions regarding the superiority of EPCS over conventional techniques cannot yet be drawn. Future adequately powered multicenter randomized controlled trials using standardized surgical techniques, perioperative protocols, and outcome definitions are needed to confirm these findings, evaluate long-term maternal and neonatal outcomes, and better define the role of EPCS and FAUCS in contemporary obstetric practice.

## Figures and Tables

**Table 1:** Search strings and number of results across databases.

PUBMED *n* = 20	((“extraperitoneal cesarean section”[Title/Abstract] OR “French Ambulatory Cesarean Section”[Title/Abstract] OR “French Ambulatory Cesarean”[Title/Abstract] OR “FAUCS”[Title/Abstract])) AND ((“safety”[MeSH Terms] OR “safety”[Title/Abstract]) OR (“complications”[MeSH Terms] OR “complications”[Title/Abstract]) OR (“mortality”[MeSH Terms] OR “mortality”[Title/Abstract]) OR (“maternal outcomes”[Title/Abstract]) OR (“neonatal outcomes”[Title/Abstract])))
SCOPUS *n* = 51	(TITLE-ABS-KEY (“extraperitoneal cesarean section” OR “French Ambulatory Cesarean Section” OR “French Ambulatory Cesarean” OR “FAUCS”)) AND (TITLE-ABS-KEY (“safety” OR “complications” OR “mortality” OR “maternal outcomes” OR “neonatal outcomes”))
Web of Science *n* = 18	TS = (“extraperitoneal cesarean section” OR “French Ambulatory Cesarean Section” OR “French Ambulatory Cesarean” OR “FAUCS” OR “extraperitoneal c-section” OR “ambulatory cesarean section” OR “outpatient cesarean section”) AND TS = (“safety” OR “complications” OR “mortality” OR “maternal outcomes” OR “neonatal outcomes” OR “adverse events” OR “recovery time” OR “postoperative outcomes”)

**Table 2:** Summary of studies comparing EPCS and TCS [9,11,12,23].

Study	Year	Country	No. of Patients (EPCS/TCS)	Method	Primary Outcome	Secondary Outcomes
Khoiwal et al. [11]	2023	India	34/34	Open-label randomized controlled trial	Fetus delivery time	Total operation time, blood loss, pain level, return of bowel function, requirement of injectable antibiotics and analgesics, Apgar score, hemoglobin difference
Tappauf et al. [9]	2013	Austria	27/27	Prospective, randomized, single (patient) blinded	Maximum abdominal pain on postoperative day 1	Analgesic requirements, intraoperative nausea, postoperative shoulder pain, urogenital distress, urinary tract infection, bowel dysfunction at discharge from hospital, long-term follow-up at 6 weeks, urogenital dysfunction, need for analgesics, overall satisfaction
Yapca et al. [23]	2020	Turkey	105/105	Randomized clinical trial	Skin incision-to-delivery time	Total operation time, intraoperative nausea, gag reflex, vomiting, pain and anxiety for those receiving regional anesthesia, pain level, change in hemoglobin, postoperative analgesic requirements, nausea, vomiting and shoulder pain, urogenital distress, time until gas passage, neonatal outcome
Bacić et al. [12]	2024	Croatia	51/49	Retrospective case–control	Not stated	Total operation time, hemoglobin, hematocrit, leucocytes, C-reactive Protein, surgical site infection (endometritis + wound infection), febrility Tax > 38 °C, Apgar score, bilirubin levels on the third day, pain level, pain medication, bowel movement (at first, second and third day)

EPCS: Extraperitoneal Cesarean Section, TCS: Transperitoneal Cesarean Section.

**Table 3:** Comparison of outcomes between EPCS and TCS across RCT studies [9,11,23].

Outcome		Khoiwal et al. [11]	Tappauf et al. [9]	Yapca et al. [23]	General Conclusion
Time to fetal delivery (minutes) *	EPCS	14.26 ± 1.26	4 (3.25–6.00)	3.9 (2.1–7.3)	TCS is generally associated with faster fetal delivery.
	TCS	9.38 ± 1.83	3 (3.00–4.50)	4.2 (1.9–8.2)	
		*p* < 0.001	*p* = 0.053	*p* = 0.065	
Total operation time (minutes)	EPCS	63.24 ± 12.74	28.9 ± 3.9	26.9 (17.2–41.3)	EPCS is associated with shorter or comparable total operation time.
	TCS	57.41 ± 8.62	34.7 ± 10.5	30 (19.9–75.8)	
		*p* = 0.027	*p* = 0.012	*p* = 0.001	
Blood loss (mL)	EPCS	733.82 ± 219.06	Not reported	Not reported	No significant difference in blood loss.
	TCS	694.12 ± 351.57			
		*p* = 0.063			
Return of bowel function **	EPCS	Faster	Not reported in terms of time	Faster (time to gas passage)	EPCS is associated with faster return of bowel function.
	TCS	Slower		Slower	
		*p* < 0.001		*p* = 0.001	
Apgar score (1 min)	EPCS	7.65 ± 0.77	9 (9–9)	9 (0–10)	No significant difference in 1 min Apgar scores.
	TCS	7.38 ± 1.52	9 (9–9)	9 (2–10)	
		*p* = 0.604	*p* = 0.852	*p* = 0.233	
Apgar score (5 min)	EPCS	8.88 ± 0.33	10 (9.25–10)	9 (0–10)	No significant difference in 5 min Apgar scores.
	TCS	8.76 ± 0.65	10 (10–10)	9 (2–10)	
		*p* = 0.639	*p* = 0.545	*p* = 0.867	
Umbilical cord pH	EPCS	Not reported	7.27 ± 0.72	7.31 (7.11–7.43)	No significant difference in umbilical cord pH.
	TCS		7.30 ± 0.05	7.3 (7.16–7.5)	
			*p* = 0.080	*p* = 0.101	
Change in hemoglobin (g/dL)	EPCS	1.04 ± 0.51	0.75 ± 0.72	2 ± 1	EPCS is associated with lower hemoglobin change in some studies.
	TCS	1.40 ± 0.40	1.05 ± 0.85	2 ± 1	
		*p* = 0.001	*p* = 0.176	*p* = 0.220	
Length of hospital stay (days)	EPCS	4.15 ± 0.89	4 (4–5)	2 (1–6)	EPCS is associated with shorter or comparable length of hospital stay.
	TCS	4.82 ± 1.34	4 (4–4)	2 (1–7)	
		*p* = 0.027	*p* = 0.082	*p* = 0.165	

* from skin incision; ** do not specify how this parameter was measured; EPCS: Extraperitoneal Cesarean Section, TCS: Transperitoneal Cesarean Section.

**Table 4:** Summary of studies analyzing FAUCS as a study group [13,22,24,25,26,27].

Study	Year	FAUCS	Control Group	Methods	Outcomes
Hendler et al. [26]	2024	*n* = 810	TCS *n* = 217	Retrospective cohort study	Total operation time, time from skin incision to fetal extraction, maternal surgical complications (postpartum bleeding, bladder injury, bowel injury, need for blood transfusion, repeat laparotomy), neonatal outcomes (umbilical cord pH, neonatal trauma, NICU admission), changes in hemoglobin levels, pain medication requirements after the first 24 h, length of hospital stay
Mustafa et al. [27]	2023	*n* = 50	No	Retrospective descriptive study	Total operation time, time from skin incision to fetal extraction, blood loss, bladder injury, pain level, pain medication, time to spontaneous urination, need for urinary tract catheter, postoperative hemoglobin, postoperative anemia, blood transfusion, length of hospital stay, endometritis, incision hematoma, maternal satisfaction
Ami et al. [13]	2017	*n* = 3441	No	Retrospective descriptive study	Total operation time, length of hospital stay, bladder injury, hematoma in the retropubic space, parietal abscesses, pain medication
Dimassi et al. [24]	2020	*n* = 60	MLCS *n* = 52	Prospective cohort study	Total operation time, time from skin incision to fetal extraction, type of instruments used to extract the fetus, blood loss, maternal surgical complications (hemorrhage, bladder injury), pain level (every 6 h for 24 h), pain medication, time to first time standing up, time to full meal, time to urinate, time to hospital discharge, time to mother’s autonomy (recovery of physiological functions), neonatal outcomes (Apgar score at 5 min)
Dimassi et al. [22]	2021	*n* = 50	MLCS *n* = 50	Randomized clinical trial	Total operation time, instruments used to extract the fetus, blood loss, pain level, combined pain and medication scores (PAMS), time to mother’s autonomy (recovery of physiological functions), length of hospital stay, neonatal outcomes (Apgar score at 1, 3, 5, 10 min, NICU admission, umbilical cord pH).
Wolder et al. [25]	2025	*n* = 30	TCS *n* = 36	Prospective cohort study	Surgery duration, blood loss, time to mobilization, oral food intake, and initiation of breastfeeding. Neonatal outcomes included birth weight, Apgar scores, umbilical cord arterial pH

TCS: Transperitoneal Cesarean Section, FAUCS: French Ambulatory Cesarean Section, MLCS: Misgav Ladach Cesarean Section, NICU: Neonatal Intensive Care Unit.

## Data Availability

The data supporting the findings of this study are derived from published articles included in the systematic review. All relevant data are contained within the article and its Supplementary Materials. No new datasets were generated during the current study.

## Supplementary Materials

The following supporting information can be downloaded at https://www.mdpi.com/article/10.3390/jcm15166153/s1: Table S1: PRISMA checklist for systematic review (Reference [28] is cited in the Supplementary Materials).
